# Isocitrate dehydrogenase (IDH) status prediction in histopathology images of gliomas using deep learning

**DOI:** 10.1038/s41598-020-64588-y

**Published:** 2020-05-07

**Authors:** Sidong Liu, Zubair Shah, Aydin Sav, Carlo Russo, Shlomo Berkovsky, Yi Qian, Enrico Coiera, Antonio Di Ieva

**Affiliations:** 1 Department of Clinical Medicine, Faculty of Medicine, Health and Human Sciences, Macquarie University, Sydney, Australia; 2 Computational NeuroSurgery (CNS) Lab, Department of Clinical Medicine, Faculty of Medicine, Health and Human Sciences, Macquarie University, Sydney, Australia; 3 Centre for Health Informatics, Australian Institute of Health Innovation, Faculty of Medicine, Health and Human Sciences, Macquarie University, Sydney, Australia; 4 College of Science and Engineering, Hamad Bin Khalifa University, Doha, Qatar; 5 Department of Pathology, Yeditepe University, School of Medicine, Istanbul, Turkey; 6 Department of Biomedical Sciences, Faculty of Medicine, Health and Human Sciences, Macquarie University, Sydney, Australia

**Keywords:** Diagnostic markers, Translational research, CNS cancer

## Abstract

Mutations in isocitrate dehydrogenase genes *IDH1* and *IDH2* are frequently found in diffuse and anaplastic astrocytic and oligodendroglial tumours as well as in secondary glioblastomas. As IDH is a very important prognostic, diagnostic and therapeutic biomarker for glioma, it is of paramount importance to determine its mutational status. The haematoxylin and eosin (H&E) staining is a valuable tool in precision oncology as it guides histopathology-based diagnosis and proceeding patient’s treatment. However, H&E staining alone does not determine the IDH mutational status of a tumour. Deep learning methods applied to MRI data have been demonstrated to be a useful tool in IDH status prediction, however the effectiveness of deep learning on H&E slides in the clinical setting has not been investigated so far. Furthermore, the performance of deep learning methods in medical imaging has been practically limited by small sample sizes currently available. Here we propose a data augmentation method based on the Generative Adversarial Networks (GAN) deep learning methodology, to improve the prediction performance of IDH mutational status using H&E slides. The H&E slides were acquired from 266 grade II-IV glioma patients from a mixture of public and private databases, including 130 IDH-wildtype and 136 IDH-mutant patients. A baseline deep learning model without data augmentation achieved an accuracy of 0.794 (AUC = 0.920). With GAN-based data augmentation, the accuracy of the IDH mutational status prediction was improved to 0.853 (AUC = 0.927) when the 3,000 GAN generated training samples were added to the original training set (24,000 samples). By integrating also patients’ age into the model, the accuracy improved further to 0.882 (AUC = 0.931). Our findings show that deep learning methodology, enhanced by GAN data augmentation, can support physicians in gliomas’ IDH status prediction.

## Introduction

Mutations in isocitrate dehydrogenase genes *IDH1* and *IDH2* are frequently found in diffuse and anaplastic astrocytic and oligodendroglial tumours as well as in secondary glioblastomas^1^. The analysis of the mutation in the *IDH1* and *IDH2* genes provides important diagnostic and prognostic information in patients affected by gliomas^2,3^. Moreover, knowledge of the IDH status might also be associated with the predicted response to anti-IDH treatment or vaccines^4–8^, making IDH an important therapeutic biomarker for individualised treatment as well. Recent studies suggest that IDH mutations occur in the early stage of gliomagenesis and play a critical role in glioma development^9,10^. IDH mutation is more commonly seen in lower grade gliomas (81%), including astrocytoma (69%), oligoastrocytoma (87%) and oligodendroglioma (89%); whereas the frequency of IDH mutation is substantially lower in primary glioblastoma (~8%)^1,9^. IDH is a very important prognostic, diagnostic and therapeutic biomarker for glioma, and triggered the integrated genomic-histological characterization of brain tumours proposed in the 2016 World Health Organization (WHO) classification system^1^. Recently, some studies have shown IDH mutational status may be predicted using neuroimaging with good accuracy (between 78.2% and 92.8%)^11–20^, and also with very good diagnostic performance when using 2-hydroxyglutarate MR spectroscopy (2HG-MRS, with a pooled 91% sensitivity and 95% specificity)^21,22^. However, neuroimaging is not yet state-of-the-art in detecting IDH mutations in glioma, which is one of the reasons tumour sampling is often still necessary, also because surgical resection/debulking is part of the current mainstay of treatment^23^. Following surgical sampling, the current gold standard to detect the mutation is immunohistochemistry (using R132H antibody)^24^ and/or genetic sequencing of the fresh sample^1^. Both can be difficult and expensive, and many hospitals are not able to perform these techniques; instead outsourcing the analysis or labelling the patients as ‘IDH non-otherwise specified (IDH NOS)’.

The haematoxylin and eosin (H&E) stain in histopathology is a valuable tool for precision oncology and is used in assisting the diagnosis of glioma and other tumours. However, pathologists’ visual interpretation of H&E-stained slides does not allow for the determination of the IDH mutational status. The effectiveness of deep learning in classification and mutation prediction of H&E slides has recently been explored for non-small cell lung cancer^25^ and in virtual histological staining of unlabelled tissue images^26^. Its use in gliomas has not been fully investigated^27,28^. To the best of our knowledge, there exists only one study that used deep learning for IDH mutational status prediction based on the histopathology images, with an accuracy of 0.79 and area under the curve (AUC) of 0.86 (ref. ^29^). However, it is not clear how the patients were selected in that study. Furthermore, the performance of previous deep learning methods on either MRI or H&E slides remains unclear because of the small sample sizes and unbalanced sample distributions in past studies^11–20^.

In this study, we propose a deep learning-based model for histopathological image classification. This model is enhanced by a data augmentation method based on Generative Adversarial Network (GAN)^30^. GAN provides a new opportunity to alleviate the problem related to relatively small samples by transforming the discrete distribution of training samples into a new continuous distribution and by generating synthetic samples with high fidelity according to the estimated new sample distribution^30^. In a recent study, it has been demonstrated that GAN augmentation can effectively improve the performance of the deep learning models in brain lesion segmentation^31^.

The contribution of this work is two-fold. We first demonstrate that deep learning is a useful and accurate tool in differentiating IDH mutation from IDH-wildtype gliomas based on histopathology images. Furthermore, we demonstrate that GAN-based data augmentation may further assist in histopathology image classification.

## Results

### A deep-learning framework for IDH status prediction in gliomas histopathology slides

In this study, 200 patients were randomly selected from the glioma cohorts of The Cancer Genome Atlas (TCGA)^32^ and another cohort of 66 patients were recruited at a local hospital (see methods). We randomly divided this combined dataset into training, validation and test sets using a 6:1:1 ratio. ResNet50, which is a convolution neural network (CNN) approach allowing us to tack multiple convolutional layers with nonlinear activation functions and low network complexity, was used, aimed to develop hierarchical representations of imaging data^33,34^. The baseline deep learning model based on ResNet50 without data augmentation (24,000 image samples) achieved the accuracy of 0.765 (AUC = 0.823) on the validation set, and the accuracy of 0.794 (AUC = 0.920) on the test set. These results demonstrate that CNNs can effectively identify the IDH mutational status.

**Figure 1:**
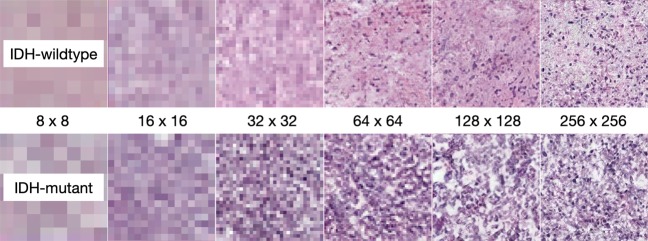
Samples of the GAN synthesised images from coarse to fine scales.

With GAN-based data augmentation, the accuracy of the IDH mutational status prediction was improved to 0.853 (AUC = 0.868) on the validation set, and 0.853 (AUC = 0.927) on the test set when 3,000 GAN generated training samples were added to the original training set (24,000 samples). We further integrated age into the image-based classification using a logistic regression classifier, which improved the performance to an accuracy of 0.853 (AUC = 0.882) on the validation set, and an accuracy of 0.882 (AUC = 0.931) on the test set. Thus, we observe that the augmentation of patient images with GAN-generated ones further improves the performance. Figure 1 shows an example of the images synthesised by means of the GAN-based approach.

**Figure 2:**
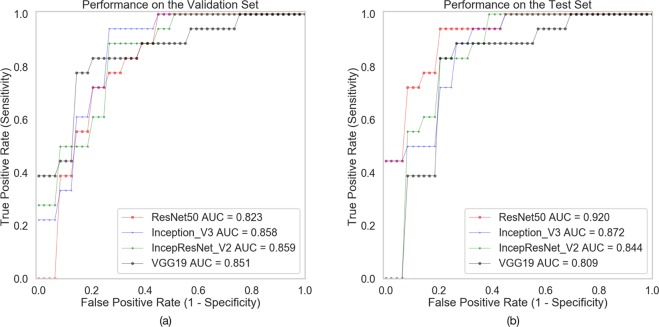
Receiver Operating Characteristics (ROC) curves of different DNN models on the validation set (**a**) and test set (**b**).

In Table 1 and Fig. 2, the classification performance of different deep neural network (DNN) models is shown, comparing sensitivity, specificity, accuracy and AUC.

**Table 1:** Classification performance of different DNN models on the validation and test sets.

	DNN models	Sensitivity	Specificity	Accuracy	AUC
On validation set	ResNet50	0.722	**0.813**	**0.765**	0.823
	Inception_V3	0.722	0.750	0.735	0.858
	IncepResNet_V2	**0.833**	0.688	**0.765**	**0.859**
	VGG19	**0.833**	0.688	**0.765**	0.851
On test set	ResNet50	0.778	**0.813**	**0.794**	**0.920**
	Inception_V3	0.778	0.750	0.765	0.872
	IncepResNet_V2	**0.833**	0.750	**0.794**	0.844
	VGG19	0.778	**0.813**	**0.794**	0.809

**Figure 3:**
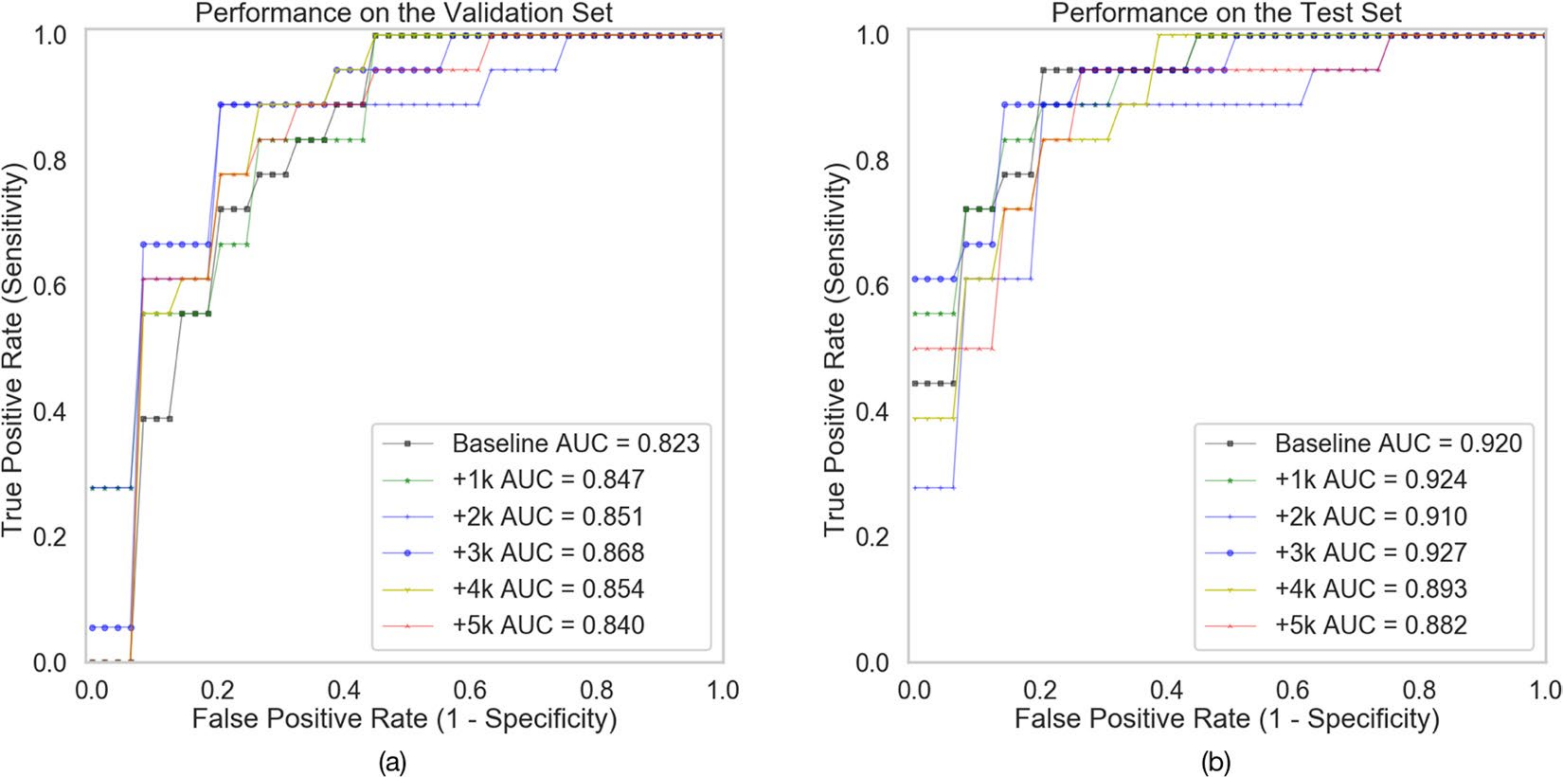
ROC curves of the models with different number of GAN generated image samples on the validation set (**a**) and test set (**b**).

The GAN-based data augmentation allowed an increase in the classification performance when a max number of 3,000 images were synthesised, with the accuracy decreasing afterwards (classification performance with data augmentation shown in Table 2 and Fig. 3).

**Table 2:** Classification performance of ResNet50 on the test set with different numbers of GAN-augmented training samples. In bold, the highest values reached by GAN-based augmentation of the dataset to 3,000 synthetic images (12.5% addition to the original training set).

	Samples	Sensitivity	Specificity	Accuracy	AUC
On validation set	Baseline	0.722	0.813	0.765	0.823
	+1 K (+4.2%)	0.778	0.750	0.765	0.847
	+2 K ( + 8.3%)	0.722	**0.813**	0.765	0.851
	+3 K (12.5%)	**0.889**	**0.813**	**0.853**	**0.868**
	+4 K (+16.7%)	0.778	**0.813**	0.794	0.854
	+5 K (+20.8%)	0.833	0.750	0.794	0.840
On test set	Baseline	0.778	0.813	0.794	0.920
	+1 K (+4.2%)	**0.889**	0.813	**0.853**	0.924
	+2 K (+8.3%)	0.833	**0.875**	**0.853**	0.910
	+3 K (12.5%)	**0.889**	0.813	**0.853**	**0.927**
	+4 K (+16.7%)	0.778	0.813	0.794	0.893
	+5 K (+20.8%)	0.778	0.813	0.794	0.882

### Classification performance with data augmentation and age

**Figure 4:**
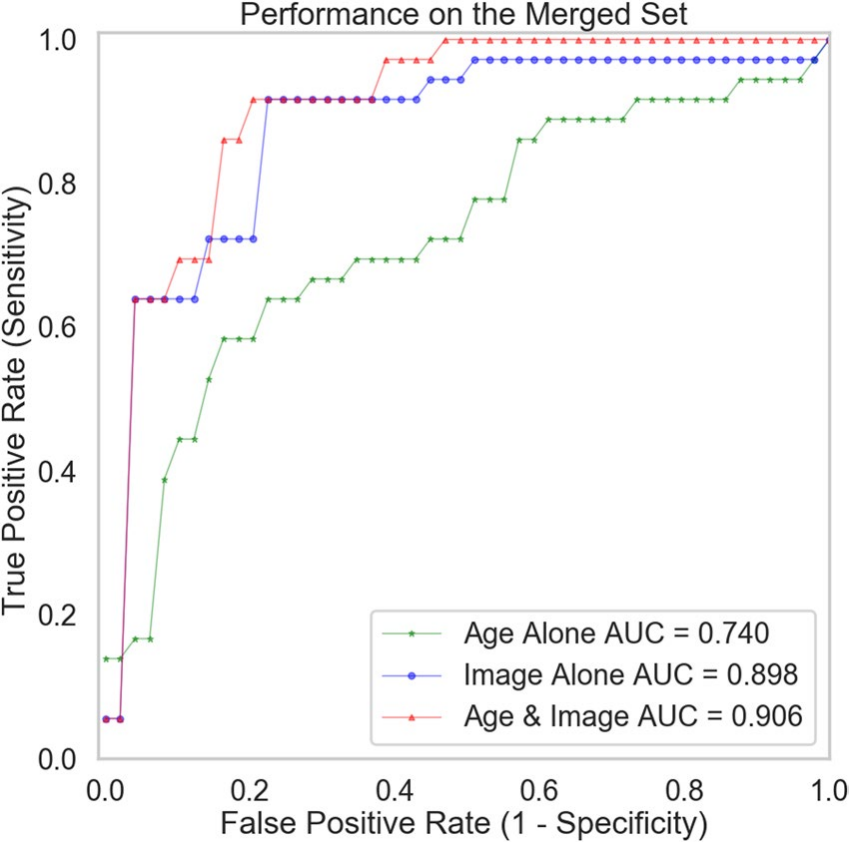
ROC curves of the models with different predictors, including age, image, and age and image combined.

It has previously been demonstrated that age is an important factor in IDH status prediction^1^. A multivariate model has shown that, in the case of a negative R132H-IDH immunohistochemistry in a primary glioblastoma, the probability to have an alternative IDH mutation is <6% in a 50-year old patient and <1% in patients older than 54, and, pragmatically, even in the absence of IDH sequencing, the labelling of such patients as “IDH-wildtype” is reasonable^35^. We therefore modelled the age distribution of the IDH-mutant and IDH-wildtype patients using a Gaussian discriminant analysis (GDA) model, and the age-based prediction was then combined with the image-based prediction using a logistic regression classifier, which further improved the performance to an accuracy of 0.853 (AUC = 0.882) on the validation set, and an accuracy of 0.882 (AUC = 0.931) on the test set (Table 3 and Fig. 4). These results show that, although age-based classification is inferior to an image-based one, their combination achieves the best performance.

**Table 3:** Classification performance of using different predictors, including age, image (based on the model trained on 3,000 GAN samples), and age and image combined.

	Predictor	Sensitivity	Specificity	Accuracy	AUC
On validation set	Age	0.667	0.563	0.618	0.724
	Image	**0.889**	**0.813**	**0.853**	0.868
	Age & image	**0.889**	**0.813**	**0.853**	**0.882**
On test set	Age	0.722	0.750	0.735	0.786
	Image	0.889	**0.813**	0.853	0.927
	Age & image	**0.944**	**0.813**	**0.882**	**0.931**

### Performance evaluation on different age groups

The patients were stratified into two age groups, i.e., younger than 55 (Group I), and 55 or older (Group II), aligned with previous studies^35^. Table 4 shows the classification results in each age group.

**Table 4:** Performance on different age groups.

	Age Group	Group I: < 55	Group II: ≥ 55
Number of patients (mutant: wildtype)	Total	44 (28:17)	24 (8:16)
Error incidences (mutant: wildtype)	Age	14 (3:11)	8 (8:0)
	Image	8 (4:4)	1 (0:1)
	Age & Image	7 (3:4)	1 (0:1)
Accuracy	Age	0.682	0.667
	Image	0.818	**0.958**
	Age & Image	**0.841**	**0.958**

We found that (1) age-based prediction alone is inaccurate in both groups, and worse in the ≥55 group with an accuracy of 0.667; (2) Image-based predictions outperform age-based predictions (although we acknowledge that the sample sizes were small); and (3) it was challenging to classify Group I patients for the individual age- and image-based models, yet their combination performed better than either predictor alone. This further refines our observation regarding the performance of the combined age- and image-based model.

## Discussion

We proposed a novel deep learning-based method to predict the *IDH1/2* mutation status in a glioma, even prior to IDH1 immunohistochemistry (which is typically performed using an antibody against the R132H mutation) and/or genetic sequencing. We demonstrated its ability to predict IDH mutation status in H&E stained slides. In order to overcome the limitations of a small sample size for machine training purposes, we also introduced a GAN-based pipeline for data augmentation, aimed to create synthetic histopathology images of gliomas. By applying a baseline deep learning model without data augmentation, a close to 80% accuracy in predicting the IDH status on H&E histopathological images was achieved, which increased to about 85% with a GAN-based data augmentation technique, when 3,000 GAN generated training image samples were added to the training set. Such accuracy was further increased to about 87% when patient’s age was integrated with the image-based classification, and, specifically, ~96% in the group of patients older than 55 years.

As it is not possible by expert pathologists to classify a glioma as IDH-mutant or IDH-wildtype on the basis of the simple visual inspection of the H&E stained histological specimen of glioma, these are encouraging results. The deep learning method could be used to augment a physician’s diagnostic and prognostic evaluation and improve decision-making in regards to treatment of patients affected by glioma^36^. Our study represents a first step in validation, and more studies to test generalisability are needed, in order to determine the impact on patients’ outcome^37^.

The training phase of the deep neural network methodology and, above all, the training of the GAN models for image synthesis, can take a considerable amount of time on a desktop personal computer, although the use of graphics processing units (GPUs) speeds up the procedure. However, once the models are fully trained, it only takes a few seconds to generate the synthesized images and to classify the test images. The pre-processing (image tiling, tissue segmentation, colour conversion, histogram equalization, gamma adjustment) of a single whole-slide image may take a few minutes, depending on the image size. Overall, the data processing is still shorter than any immunohistochemistry-based histopathology techniques, not to mention the long time (and costs) required for genetic sequencing. Furthermore, the trained DNN models can be seamlessly deployed to other systems with compatible settings, which further enhances the clinical applicability of the proposed method.

In actual clinical practice, a glioma should be classified as wildtype when both R132H-IDH immunohistochemistry and subsequent IDH1/2 sequencing revel wildtype sequences at *IDH1* codon 132 and *IDH2* codon 172^1^. This is not always possible, and the definition NOS (not otherwise specified), in which the pathology assessment of IDH mutation is inconclusive or unavailable is the consequence, leaves doctors, patients and family uncertain of the precise diagnosis, prognosis and therapeutic options. The utilisation of deep learning for analysis of histopathology slides of gliomas could potentially decrease the rate of the diagnosis of glioma NOS. The standardisation of the technique (and eventually the centralisation of the post-processing analysis), associated with the patient’s age and eventually corroborated by the use of pre-operative neuroimaging (including 2HG-MRS technique)^20,38^, will eventually make performing further histopathological analyses less necessary (or unnecessary, in selected cases, such as older patients), saving time and reducing the costs related to patients’ diagnosis and care. In the future, a pathologist could take a photo of the processed H&E slide and submit it to a system trained according to our method, getting the IDH status prediction back in a few minutes, according to which further tests, whenever necessary, can be arranged. A further future perspective relates to a practical aspect occurring during glioma surgery: considering the increasing evidence on the positive relationship between the degree of surgical resection and extent of the survival in patients affected by gliomas harbouring IDH mutations (i.e., IDH-mutant gliomas patients have a better prognosis when the resection is total or even supra-total vs. the ones who undergo a sub-total/partial resection)^23,39–41^, the surgical approach could be tailored intra-operatively according to the IDH status findings. Indeed, the surgical sample might be stained with H&E and, avoiding the delays related to the immunohistochemistry against the IDH antibody, the image of the slide could be computationally processed in a few minutes according to our methodology. A prediction of the presence of IDH mutation in the sample might warrant a more extended surgical resection, aimed at a complete removal of the tumour, in order to improve patient’s prognosis, whilst the presence of an IDH-wildtype genotype would suggest a sub-total/partial but neurologically safer resection, leaving the post-operative management of the tumour remnant to the radiochemotherapy.

In our study, the relatively small sample size problem of gliomas has been minimised by the use of the recent AI-technique called GAN^30^. GAN models are designed to learn the hidden patterns of the available samples and produce a smoother distribution of the samples. Therefore, with the help of these GAN synthetic samples, a discriminative model can define a smoother decision ‘boundary’ between different classes. It is interesting to note that the augmentation of the dataset to 3,000 images increased the prediction accuracy of the model, although we found that when the training data increased to 4,000 and 5,000 images, the performance decreased. This implies that the increasing the number of GAN-synthesised samples may increase the chances to cover the hidden patterns of the samples, but in the meantime, produce low fidelity images and induce bias to the classification model, therefore a cut-off to the new generated sample should be identified, as we did here.

By standardising deep learning image analysis of brain tumour histopathology samples, computational analysis should be able to assist pathologists and, in some cases, possibly replace laboratory (pathology) techniques, to eventually become the diagnostic and prognostic gold standard for brain lesion characterisation. This might speed up decision-making and allow a better and faster flow of reliable information among neuro-oncology multidisciplinary teams^36^. Although our findings will need broader validation on larger multi-centric datasets and a clear translation into the clinical setting, it is clear that a validated AI-based pipeline used to merge clinical information (e.g., age) with pre-operative neuroimaging (e.g., deep learning analysis of MRI, and/or magnetic resonance spectroscopy) and post-operative histopathology feature extraction and pattern recognition will eventually translate itself in a more precise diagnosis and improved prognostic stratification and decision-making for better patient care and outcome.

As a proof of concept, this study has a few limitations. First, only 200 patients were selected from the TCGA cohort and combined with a second cohort of 66 patients retrospectively selected from a local hospital. Larger sample size would help train more accurate and robust classifiers as well as produce reliable performance estimate. As demonstrated in Table A1 and Figure A1 (Appendix A), when ResNet50 was trained and tested using the entire TCGA cohort of 921 patients, its performance was improved to an accuracy of 0.846 and AUC of 0.929 on the validation set, and an accuracy of 0.87 and AUC of 0.938 on the test set. However, such large datasets are not always available for the same type of analysis. As one of the contributions of this work, we hereby demonstrated that GAN might be a feasible solution to the small sample-size problems, regardless the sample-size itself.

Furthermore, the DNN models were not tested separately on IDH1 or IDH2, or any sub-types of IDH1 in this study. We will further investigate these aspects in the future work, although, in the actual neuro-oncology scenario, the differentiation of the two subgroups lacks clinical implications. In addition, the frequency of IDH mutation strongly correlates with the tumor type, *i.e*., IDH mutations exist at a much higher frequency in lower grade gliomas than glioblastomas; therefore, there is a need to remove tumor type as a confounding factor in IDH mutation status. Ideally, we should have the same number of IDH-wildtype and mutant samples in each tumor type; however, it may greatly reduce the samples that can be used. The trade-off between a balanced dataset and the number of available samples will remain an open question, but this problem will be alleviated as larger dataset become available.

## Methods

### Histopathology image dataset

We randomly selected 200 patients from The Cancer Genome Atlas (TCGA) data portal^42^, including 100 glioblastoma (GBM) patients from the TCGA GBM project, and another 100 astrocytoma and oligodendroglioma cases from the TCGA low grade glioma (LGG) project (Table 5). A detailed description of these projects can be found in ref. ^32^.

**Table 5:** The sample distribution of the TCGA patients (SD = standard deviation).

Tumour type (WHO Grade)	IDH-wildtype	IDH-mutant	Total
Glioblastoma (IV)*	70	30	100
Diffuse astrocytoma (II) #	4	14	18
Anaplastic astrocytoma (III) #	26	16	42
Oligodendroglioma (II) #	0	29	29
Anaplastic oligodendroglioma (III) #	0	11	11
Total	100	100	200
Age (mean±SD)	55.5±13.3	44.2±12.7	49.9±14.1
Gender (male:female)	58:42	59:41	117:83

*from the TCGA GBM project.

^#^from the TCGA LGG project.

A second cohort including 66 patients with gliomas (41 females, 25 males, age ranging from 13 to 78 years, average age: 49.3±12.4; WHO Grade II to IV, according to the 2016 WHO Classification of Tumours of the Central Nervous System) were retrospectively collected from a local hospital, the Yeditepe University Hospital, Istanbul, Turkey, following patients’ informed consent and approval from the Yeditepe University Clinical Research Ethics Committee. In regards to the only patient under the age of 18, informed consent was obtained from the patient’s parents.

All methods were carried out in accordance with relevant guidelines and regulations. The H&E pathological slides were acquired from the identified patients, who underwent surgery between 2016 and 2018. Tumours sections were stained with a hematoxylin and eosin solution. The dataset included 30 IDH-wildtype and 36 IDH-mutant cases (Table 6). Tumours’ histopathology diagnosis was confirmed by the local neuropathologist (A.S.). The IDH status was confirmed by immunohistochemistry and/or genetic sequencing at the same institute. Table 6 shows the sample distribution of this cohort.

**Table 6:** The sample distribution of the cases from a local hospital (SD = standard deviation).

Tumour type (WHO Grade)	IDH-wildtype	IDH-mutant	Total
Glioblastoma (IV)	21	10	31
Diffuse astrocytoma (II)	4	3	7
Anaplastic Astrocytoma (III)	4	0	4
Oligodendroglioma (II)	0	7	7
Anaplastic oligodendroglioma (III)	0	16	16
Diffuse Midline Glioma (IV)	1	0	1
Total	30	36	66
Age (mean±SD)	53.3±14.7	45.9±9.0	49.3±12.4
Gender (male:female)	14:16	11:25	25:41

We combined the two cohorts and then randomly split the dataset into training, validation and test sets in a 6:1:1 ratio. Therefore, there were 198 patients in the training set, and 34 patients in both the validation set and test set. The validation set was used during the training process to evaluate the model’s performance in each training epoch as the model’s weights were updated. In contrast, the test dataset was used to evaluate the model’s performance after the model was fully trained.

### Overview of the algorithm

**Figure 5:**
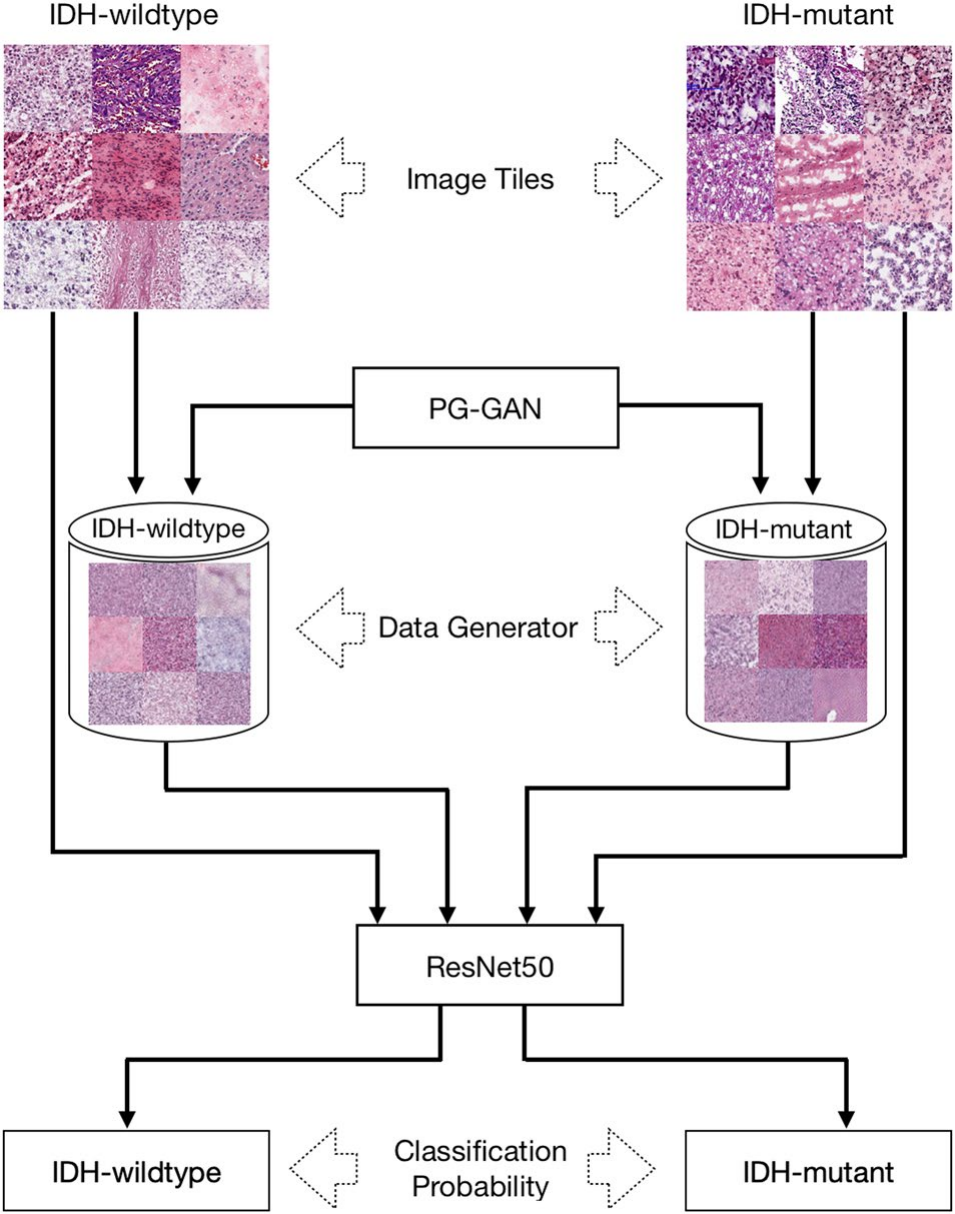
A generalized overview of the proposed method, including two GAN models for data augmentation and a ResNet50 model for image classification.

The proposed method contains two major components. First, we designed two GAN models^43^ for modelling the data distribution of the IDH-mutant and IDH-wildtype samples, respectively. Furthermore, the ResNet50 model^33^ was implemented for image classification. We then gradually fed the GAN-generated images to the ResNet50 model to enhance its performance for IDH status classification. A generalized overview of the proposed method is shown in Fig. 5.

### Image pre-processing

**Figure 6:**
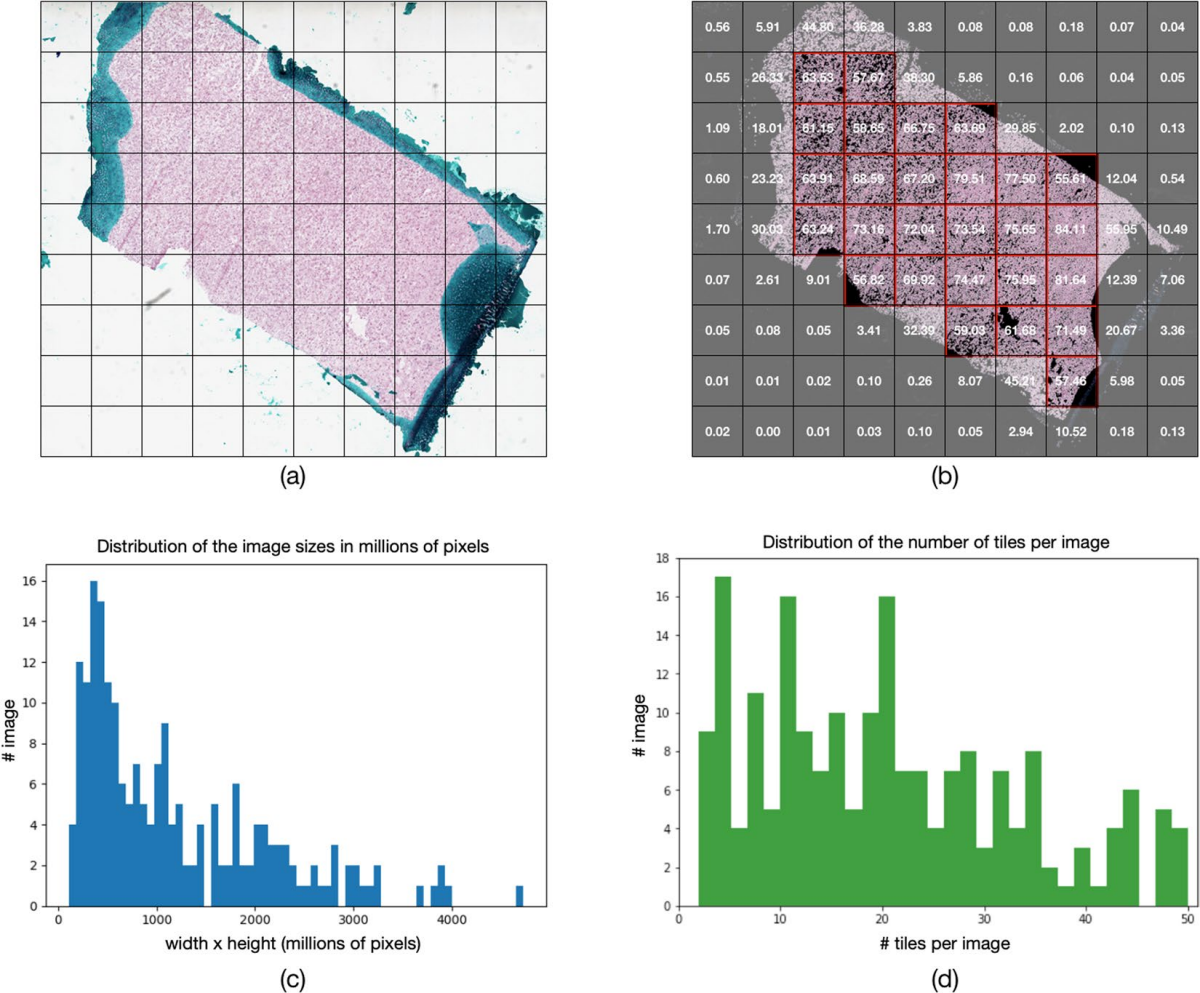
TCGA data pre-processing. (**a**) whole-slide image partition; (**b**) tissue proportion-based tile selection; (**c**) distribution of whole-slide image sizes; (**d**) distribution of number of selected tiles per image.

The TCGA whole-slide images were pre-processed using the Python Whole-Slide Image (WSI) pre-processing pipeline (https://github.com/deroneriksson/python-wsi-preprocessing). Such a pipeline is a well-established software package with Python Application Program Interface (API), which provides the flexibility to construct the own pre-processing pipeline, according to the necessities. For each TCGA whole-slide image, we first divided the slide into 1024 × 1024-pixel tiles at 10 × magnification (as shown in Fig. 6a), and then applied a sequence of image filters, including a background filter, a shadow filter, three pen marks filters and a small object filter, to the tiles for tissue segmentation. More details can be found in the online tutorial (https://github.com/deroneriksson/python-wsi-preprocessing). Only tiles that consisted of at least 50% tissue were selected for analysis. Figure 6b shows the segmented tissue and the numbers indicate tissue proportion in each tile. The TCGA whole-slide images have a wide range of sizes, as illustrated in Fig. 6c, and tens to hundreds of tiles can be extracted from each whole-slide image. To minimize the impact of the biased distribution of the number of tiles per image, we set the maximum number of tiles to be selected per slide to 50, sorted by the tissue proportion level. Figure 6d illustrates the distribution of the number of tiles extracted from the TCGA slides (4,063 image tiles in total with a median of 17 and a mean of 21 tiles per slide). For each patient in the second cohort, a neuropathologist (A.S.) cropped a single tile from the whole-slide image at the same magnification.

To prepare the training data for the two GAN models, we randomly sampled 12,000 256 × 256-pixel image patches from the IDH-mutant tiles, and another 12,000 image patches from the IDH-wildtype tiles in the training set. The RGB channels were preserved in the extracted image tiles with histogram normalization in each channel. To prepare the data for the ResNet50 model, the image patches, either extracted from the histopathology image tiles or synthesized by the two GAN models, were converted to grayscale to eliminate the heterogeneity in colour tones across the histopathology slides. Adaptive histogram equalization and gamma adjustment were then applied to every image patch to normalize the pixel intensity distribution.

### GAN for data augmentation

A GAN is composed of a Generator (G) and a Discriminator (D), where G applies transform to the input image to generate the output image which is expected to match the target image, and D compares the input image and an unknown image (either a real image from the dataset or an output image produced by G) to guess if the unknown image was generated by G. During training, D and G compete against each other, so that D can learn to detect the subtle differences between generated images and the target images, and in the same time G can learn to produce images which have high fidelity with the target images. Two GANs, namely *GAN-wildtype* and *GAN-mutant*, were trained for two groups of images in this study, as shown in Fig. 5. Each GAN was trained with 12,000 image patches extracted from the training set and learned the manifold containing the data distribution of either IDH-wildtype or IDH-mutant images. We chose the Progressive Growing of GAN (PG-GAN) model^43^ to compute the sample distribution of the data and to generate the synthetic data with training stability at large image sizes and apparent robustness to hyperparameter selection. The networks were configured to produce images with a size of 256 × 256 pixels with 6 resolution levels. We adopted the TensorFlow implementation of PGGAN^43^ and trained the data using the default parameters (available at https://github.com/tkarras/progressive_growing_of_gans/tree/master).

### ResNet50 for image classification

The ResNet architecture is designed to ease the difficulty of training deep neural networks by adding the skipping shortcut connections between one layer and a few stacked layers after that layer, to fit a residual mapping, so that the network can avoid getting saturated rapidly and the depth of the network can be increased greatly even to 1,000 layers while maintaining low complexity^33^. A few models based on the ResNet architecture (ResNet34, ResNet50, ResNet101, ResNet152) have been tested on the ImageNet dataset^44^, and the ResNet50 model is also used in medical image classification, e.g., detecting glaucomatous discs from retinal photos^45^, with human-like level performance. We used the ResNet50 model as the backbone of the method for image classification. We assigned the slide-level label to every patch and performed patch-level classification. At the patient level, the aggregated class probabilities over all image patches from the same subject were used to classify a case. The ResNet50 model built into the TensorFlow package was adopted in this study for image classification. In this study, the ImageNet pre-trained weights were used to initialize the model. A dropout layer^46^ was added on the output layer before the softmax classification layer to control overfitting. Adam optimizer^47^ was used with a batch size of 16, learning rate of $1\times {10}^{-5}$, decay rate of $1\times {10}^{-6}$, momentum of 0.9, and 100 epochs.

### Combining age- and image-based prediction

We first modelled the age distribution of the IDH-mutant and IDH-wildtype patients using two Gaussian distribution functions, then the probability of a patient being in the IDH-mutant or IDH-wildtype class was derived using the Gaussian discriminant analysis (GDA) model. The age-based prediction was then combined with the image-based prediction using a logistic regression classifier.

### Performance evaluation

We carried out two experiments to verify the performance of the proposed data augmentation and image classification methods. To verify the performance of the ResNet50 model, in the first experiment, we compared ResNet50 with 3 other state-of-the-art image classification models, namely VGG19 (ref. ^48^), Inception_V3 (ref. ^49^) and InceptionResNet_V2 (ref. ^50^) based on the same training, validation and test sets, and the same parameter settings. To evaluate the effectiveness of GAN data augmentation, in the second experiment, we fixed the classification model to ResNet50 and gradually increased the number of synthetic samples to the training set, by 1,000 images each time, to retrain the model and test whether there was any further improvement. Two video clips of the evolution of GAN generated samples are available on YouTube (IDH-wildtype: https://youtu.be/89Y3Gsha858; IDH-mutant: https://youtu.be/3HqllstPHbY). The ResNet50 model without data augmentation was used as the baseline method. The validation and test sets remained the same in this experiment, whereas the number of GAN-generated training samples was gradually increased from 1,000 to 5,000 images. Sensitivity, specificity, accuracy and area under curve (AUC) of the receiver operating characteristic curve (ROC) were used to evaluate the classification performance.

## Supplementary information

Supplementary information

## References

[1] Louis DN (2016). The 2016 world health organization classification of tumors of the central nervous system: a summary. Acta Neuropathol..

[2] Parsons DW (2008). An integrated genomic analysis of human glioblastoma multiforme. Science.

[3] Yan H (2009). IDH1 and IDH 2 mutations in gliomas. N. Engl. J. Med.

[4] Wang F (2013). Targeted inhibition of mutant IDH2 in leukemia cells induces cellular differentiation. Science.

[5] Schumacher T (2014). A vaccine targeting mutant IDH1 induces antitumour immunity. Nature.

[6] Pusch S (2017). Pan-mutant IDH1 inhibitor BAY 1436032 for effective treatment of IDH1 mutant astrocytoma *in vivo*. Acta Neuropathol..

[7] Bunse L (2018). Suppression of antitumor T cell immunity by the oncometabolite (R)-2-hydroxyglutarate. Nat. Med.

[8] Shankar GM (2018). Genotype-targeted local therapy of glioma. Proc. Natl. Acad. Sci..

[9] Ceccarelli M (2016). Molecular profiling reveals biologically discrete subsets and pathways of progression in diffuse glioma. Cell.

[10] Cancer Genome Atlas Research Network *et al*. Comprehensive, integrative genomic analysis of diffuse lower-grade gliomas. *N. Engl. J. Med*. **372**, 2481–98 (2015). 10.1056/NEJMoa1402121 PMC4530011 26061751

[11] Zhang B (2017). Multimodal MRI features predict isocitrate dehydrogenase genotype in high-grade gliomas. Neuro. Oncol.

[12] Chang K (2018). Residual convolutional neural network for the determination of *IDH* status in low- and high-grade gliomas from MR imaging. Clin. Cancer Res..

[13] Li Z, Wang Y, Yu J, Guo Y, Cao W (2017). Deep learning based radiomics (DLR) and its usage in noninvasive IDH1 prediction for low grade glioma. Sci. Rep.

[14] Liang S (2018). Multimodal 3D DenseNet for IDH genotype prediction in gliomas. Genes (Basel)..

[15] Zhou H (2019). Machine learning reveals multimodal MRI patterns predictive of isocitrate dehydrogenase and 1p/19q status in diffuse low- and high-grade gliomas. J. Neurooncol..

[16] Choi, K. S., Choi, S. H. & Jeong, B. Prediction of IDH genotype in gliomas with dynamic susceptibility contrast perfusion MR imaging using an explainable recurrent neural network. *Neuro. Oncol*. 10.1093/neuonc/noz095 (2019). 10.1093/neuonc/noz095 PMC7594560 31127834

[17] Hyare H (2019). Modelling MR and clinical features in grade II/III astrocytomas to predict IDH mutation status. Eur. J. Radiol..

[18] Tan, Y. *et al*. A radiomics nomogram may improve the prediction of IDH genotype for astrocytoma before surgery. *Eur. Radiol*. 10.1007/s00330-019-06056-4 (2019). 10.1007/s00330-019-06056-4 30972543

[19] Lee MH (2019). Prediction of IDH1 mutation status in gliobalstoma using machine learning technique based on quantitative radiomic data. World Neurosurgery.

[20] Di Ieva A (2020). Magnetic resonance spectroscopic assessment of isocitrate dehydrogenase status in gliomas: The new frontiers of spectrobiopsy in neurodiagnostics. World Neurosurgery.

[21] Choi C (2012). 2-hydroxyglutarate detection by magnetic resonance spectroscopy in IDH-mutated patients with gliomas. Nat. Med.

[22] Suh CH, Kim HS, Jung SC, Choi CG, Kim SJ (2018). 2-Hydroxyglutarate MR spectroscopy for prediction of isocitrate dehydrogenase mutant glioma: a systemic review and meta-analysis using individual patient data. Neuro. Oncol.

[23] Sanai N, Berger MS (2018). Surgical oncology for gliomas: the state of the art. Nat. Rev. Clin. Oncol..

[24] Capper D, Zentgraf H, Balss J, Hartmann C, von Deimling A (2009). Monoclonal antibody specific for IDH1 R132H mutation. Acta Neuropathol..

[25] Coudray N (2018). Classification and mutation prediction from non-small cell lung cancer histopathology images using deep learning. Nat. Med.

[26] Rivenson, Y. *et al*. Virtual histological staining of unlabelled tissue-autofluorescence images via deep learning. *Nat. Biomed. Eng*. 10.1038/s41551-019-0362-y (2019). 10.1038/s41551-019-0362-y 31142829

[27] Ertosun, M. G. & Rubin, D. L. Automated grading of gliomas using deep learning in digital pathology images: A modular approach with ensemble of convolutional neural networks. *Annu. Symp. Proceedings* **2015**, 1899–908 (2015). PMC4765616 26958289

[28] Yonekura A, Kawanaka H, Prasath VBS, Aronow BJ, Takase H (2018). Automatic disease stage classification of glioblastoma multiforme histopathological images using deep convolutional neural network. Biomed. Eng. Lett.

[29] Momeni, A., Thibault, M. & Gevaert, O. Deep recurrent attention models for histopathological image analysis. *bioRxiv* 438341 10.1101/438341 (2018).

[30] Goodfellow, I. J. *et al*. Generative Adversarial Networks. *Advances in Neural Information Processing Systems* **27**, 2672–2680 Available at, https://arxiv.org/abs/1406.2661 (2014).

[31] Bowles, C. *et al*. GAN Augmentation: Augmenting training data using generative adversarial networks. Available at: https://arxiv.org/abs/1810.10863 (2018).

[32] Clark K (2013). The Cancer Imaging Archive (TCIA): maintaining and operating a public information repository. J. Digit. Imaging.

[33] He, K., Zhang, X., Ren, S. & Sun, J. Deep residual learning for image recognition. Available at: https://arxiv.org/abs/1512.03385 (2015).

[34] LeCun Y, Bengio Y, Hinton G (2015). Deep learning. Nature.

[35] Chen L (2014). Predicting the likelihood of an isocitrate dehydrogenase 1 or 2 mutation in diagnoses of infiltrative glioma. Neuro. Oncol.

[36] Di Ieva A (2019). AI-augmented multidisciplinary teams: hype or hope?. Lancet.

[37] Coiera E (2019). On algorithms, machines, and medicine. Lancet Oncol..

[38] Di Ieva A, Choi C, Magnussen JS (2018). Spectrobiopsy in neurodiagnostics: the new era. Neuroradiology.

[39] Beiko J (2014). IDH1 mutant malignant astrocytomas are more amenable to surgical resection and have a survival benefit associated with maximal surgical resection. Neuro. Oncol.

[40] Yordanova YN, Moritz-Gasser S, Duffau H (2011). Awake surgery for WHO Grade II gliomas within “noneloquent” areas in the left dominant hemisphere: toward a “supratotal” resection. J. Neurosurg..

[41] Poulen, G., Gozé, C., Rigau, V. & Duffau, H. Huge heterogeneity in survival in a subset of adult patients with resected, wild-type isocitrate dehydrogenase status, WHO grade II astrocytomas. *J. Neurosurg*. 1–10 10.3171/2017.10.JNS171825 (2018). 10.3171/2017.10.JNS171825 29676695

[42] Home | NIH National Cancer Institute - Genomic Data Commons. Available at: https://portal.gdc.cancer.gov/. (Accessed: 28th April 2019)

[43] Karras, T., Aila, T., Laine, S. & Lehtinen, J. Progressive growing of GANs for improved quality, stability, and variation. Available at, https://arxiv.org/abs/1710.10196 (2017).

[44] Russakovsky, O. *et al*. ImageNet large scale visual recognition challenge. Available at: https://arxiv.org/abs/1409.0575 (2014).

[45] Liu S (2018). A deep learning-based algorithm identifies glaucomatous discs using monoscopic fundus photographs. Ophthalmol. Glaucoma.

[46] Srivastava N, Hinton G, Krizhevsky A, Sutskever I, Salakhutdinov R (2014). Dropout: a simple way to prevent neural networks from overfitting. J. Mach. Learn. Res..

[47] Kingma, D. P. & Ba, J. Adam: A method for stochastic optimization.. Available at: https://arxiv.org/abs/1412.6980 (2014)

[48] Simonyan, K. & Zisserman, A. Very ceep convolutional networks for large-scale image recognition. Available at: https://arxiv.org/abs/1409.1556 (2014).

[49] Szegedy, C., Vanhoucke, V., Ioffe, S., Shlens, J. & Wojna, Z. Rethinking the inception architecture for computer vision. Available at: https://arxiv.org/abs/1512.00567 (2015).

[50] Szegedy, C., Ioffe, S., Vanhoucke, V. & Alemi, A. Inception-v4, Inception-ResNet and the impact of residual connections on learning. Available at: https://arxiv.org/abs/1602.07261 (2016).

