# Supplementary material for: Isocitrate dehydrogenase (IDH) status prediction in histopathology images of gliomas using deep learning
Source: Sci Rep. 2020 May 7;10:7733. doi: 10.1038/s41598-020-64588-y (PMC7206037; doi:10.1038/s41598-020-64588-y)
Supplement: Supplementary file 1 — Supplementary information [file 41598_2020_64588_MOESM1_ESM.docx]

**Isocitrate dehydrogenase (IDH) status prediction in histopathology images of gliomas using deep learning**

**Sidong Liu, Zubair Shah, Aydin Sav, Carlo Russo, Shlomo Berkovsky, Yi Qian, Enrico Coiera, Antonio Di Ieva**

**Appendix A** DNN classification performances on entire TCGA dataset

**Table A1** Classification performance of different DNN models on entire TCGA datasets.

|  | **DNN models** | Sensitivity | Specificity | Accuracy | AUC |
| --- | --- | --- | --- | --- | --- |
| On validation set | ResNet50 | 0.825 | **0.863** | **0.846** | 0.929 |
|  | Inception_V3 | 0.800 | **0.863** | 0.835 | **0.936** |
|  | IncepResNet_V2 | **0.850** | 0.843 | **0.846** | 0.912 |
|  | VGG19 | 0.800 | **0.863** | 0.835 | 0.913 |
| On test set | ResNet50 | **0.900** | 0.846 | 0.870 | 0.938 |
|  | Inception_V3 | **0.900** | 0.865 | 0.880 | 0.946 |
|  | IncepResNet_V2 | **0.900** | **0.904** | **0.902** | 0.946 |
|  | VGG19 | 0.875 | **0.904** | 0.891 | **0.949** |


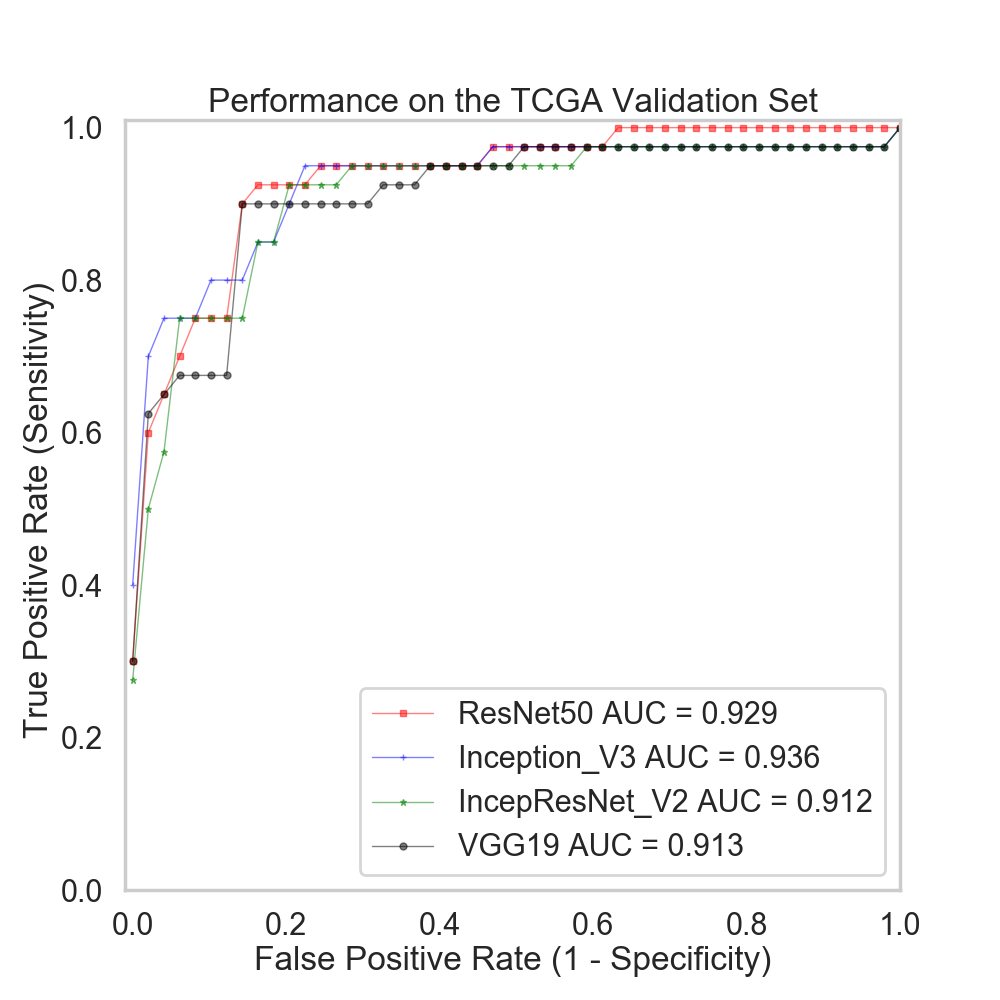

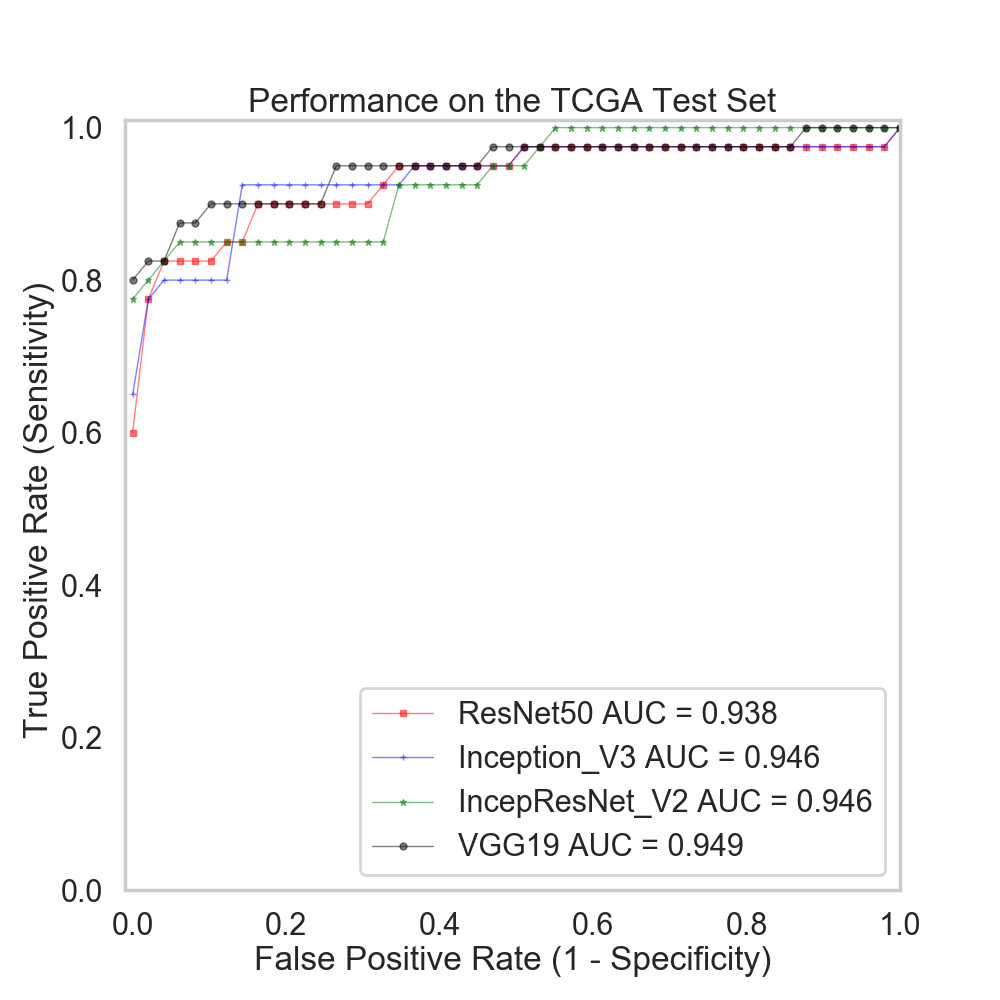


(a) (b)

**Figure A1** Receiver Operating Characteristics (ROC) curves of different DNN models on the (a) TCGA validation dataset and (b) the TCGA test set.

We evaluated the DNN models on the entire TCGA cohort of 926 patients. Five patients’ data were removed in the pre-processing stage due to the low tissue percentages in the image tiles. We randomly split the remaining 921 patients’ data into a training set (738 patients, 282,976 image samples), a validation set (91 patients, 35,728 image samples) and a test sets (92 patients, 35,904 image samples). The classification performance of different DNN models is shown in **Table A1** and **Figure A1**, comparing sensitivity, specificity, accuracy, AUC and the ROC curves.
